# Should valgus-impacted proximal humerus fractures necessarily be operated on? Radiological versus functional results

**DOI:** 10.55730/1300-0144.5674

**Published:** 2023-02-26

**Authors:** Muhlik AKYÜREK, Emre KORAMAN, Yusuf İYETİN, Mehmet AKAN

**Affiliations:** 1 Department of Orthopedics and Traumatology, Maria-Josef Hospital, Greven, Germany; 2 Department of Orthopedics and Traumatology, Faculty of Medicine, Demiroğlu Bilim University Kadıköy Florence Nightingale Hospital, İstanbul, Turkiye; 3 Department of Orthopedics and Traumatology, Pendik Bölge Hospital, İstanbul, Turkiye; 4 Department of Orthopedics and Traumatology, Faculty of Medicine, İstanbul Medeniyet University Göztepe Prof. Dr Süleyman Yalçın City Hospital, İstanbul, Turkiye

**Keywords:** Impacted valgus, functional results, radiological results, medial hinge integrity, treatment decision

## Abstract

**Background/aim:**

Impacted valgus proximal humerus fracture has been known to be challenging in terms of treatment and outcomes since it was defined. Moreover, it is a type of fracture that is difficult to treat. In addition, exact limits have not yet been determined regarding which parameters affect patients’ functional and reported outcomes. The purpose of this study was to compare the radiological results of patients with impacted valgus proximal humerus fractures treated conservatively and surgically and to evaluate the effect of these radiological parameters on functional outcomes.

**Materials and methods:**

A total of 79 patients who were treated between 2015 and 2021 with a diagnosis of impacted valgus fracture were evaluated retrospectively. Patients treated conservatively (Group 1) and surgically (Group 2) were evaluated in terms of radiological measurements (tubercle displacement (TD), cephalodiaphyseal angle (CDA), medial hinge (MH), cephaloglenoid angle (CGA), medial hinge impaction (MHI), American Shoulder and Elbow Surgeons Shoulder Score (ASES), Constant Shoulder Score, and functional outcomes (range of motion). The effect of radiological parameters on clinical outcomes was analyzed by a correlation test.

**Results:**

In the postoperative period, the ASES and Constant scores of the patients in Group 2 were significantly higher than those of the patients in Group 1. Additionally, Group 2 had better results in terms of passive extension, active internal rotation, and active/passive external rotation. Patients in both groups exhibited improvements in radiological parameters, and the correlation test showed that MH and MHI were mostly related to ASES and Constant scores.

**Conclusion:**

The monitoring and treatment of impacted valgus proximal humerus fractures remain controversial. Although radiological parameters are a guide for orthopedic surgeons, the limits have not been clearly defined. In this study, in addition to all parameters, the effect of MH and MHI on functional results was emphasized.

## 1. Introduction

Impacted valgus fracture of the proximal humerus has been previously defined and different treatment modalities have been described in the literature [1]. These fractures are distinguished from other types of complex fracture of the proximal humerus because they have a better prognosis owing to continuity of blood supply to the humeral head and fracture geometry [2,3]. However, there are still deficiencies in terms of objective criteria that will guide the treatment and prognosis apart from the surgeon’s preferences and experience when deciding on the treatment [4]. Therefore, impacted valgus fractures should be classified within themselves to clarify treatment planning. For this reason, some angular parameters have been determined to aid in the diagnosis and treatment of impacted valgus fractures [1]. These angular parameters have shown that in this fracture type the posteromedial cortex of the humeral head is mostly preserved. Therefore, the posteromedial circumflex artery is maintained. In this way, it is known that the probability of avascular necrosis of the humeral head is lower in impacted valgus fractures [5]. This is an important factor in terms of the choice of conservative treatment.

The term impacted valgus emphasizes the coronal angulation between the humeral diaphysis and humeral head and the embedding of the diaphysis in the head. However, the relationship that is impaired in impacted valgus fractures is not always only the relationship between the head of the humerus and the diaphysis [1]. Fracture of the tubercles of the proximal humerus, especially the tuberculum majus, often accompanies this fracture pattern. In addition, the medial displacement of the diaphysis and the amount of impaction are parameters that should be considered, as they may affect the clinical outcome [1,6]. Nevertheless, there are not sufficient studies on impacted valgus fractures, and surgical or conservative criteria have not yet been clearly defined [6].

The aim of the present study was to examine the effects of certain parameters on shoulder scores and range of motion (ROM) in impacted valgus fractures. The initial presentation and posthealing radiological imaging of patients with impacted valgus fractures who were treated with surgical or conservative methods were also analyzed, and their relationship with these parameters was examined by recording their results after recovery. It has been hypothesized that parameters such as tuberculum majus displacement and medial hinge amount affect clinical outcomes, and these parameters should be considered when deciding on surgical or conservative treatment in impacted valgus proximal humerus fractures.

## 2. Materials and methods

All procedures in this study that involved human participants were performed in accordance with the ethical standards of the institutional and national research committees and with the 1964 Declaration of Helsinki and its later amendments or comparable ethical standards; no animals were involved in the study. Ethics committee approval was obtained from the Clinical Research Ethics Committee of İstanbul Göztepe Prof. Dr. Süleyman Yalçın City Hospital (Date/number: 01.06.2022, 2022/0343). The aim of this retrospective, single-center, case-control study was to compare the posttreatment clinical shoulder scores and shoulder ROM of patients with impacted valgus proximal humerus fractures who were treated either conservatively or surgically. Pre- and posttreatment radiological parameters that affect the functional results between the groups were also compared. Patients who presented to the orthopedic outpatient clinic and emergency service with an impacted valgus fracture between January 2015 and January 2021 were included in the study.

The patients chosen were aged >18 years and had presented with an acute proximal humerus fracture with valgus impaction, which has been defined by many authors as a cervicodiaphyseal angle greater than 160° [7], and completed the entire treatment and follow-up process in orthopedic outpatient clinics.

The major exclusion criterion was a type of proximal humerus fracture other than impacted valgus. In addition, patients who had shoulder pain or limited ROM before the fracture due to previous shoulder pathologies such as glenohumeral arthritis or rotator cuff tears were excluded from the study. The presence of prefracture shoulder pathologies in the patients was determined by obtaining their medical history. Furthermore, patients who did not undergo a computed tomography (CT) scan at their initial or final admission and those who could not be contacted during their follow-up were excluded from the study.

**Figure 1:**
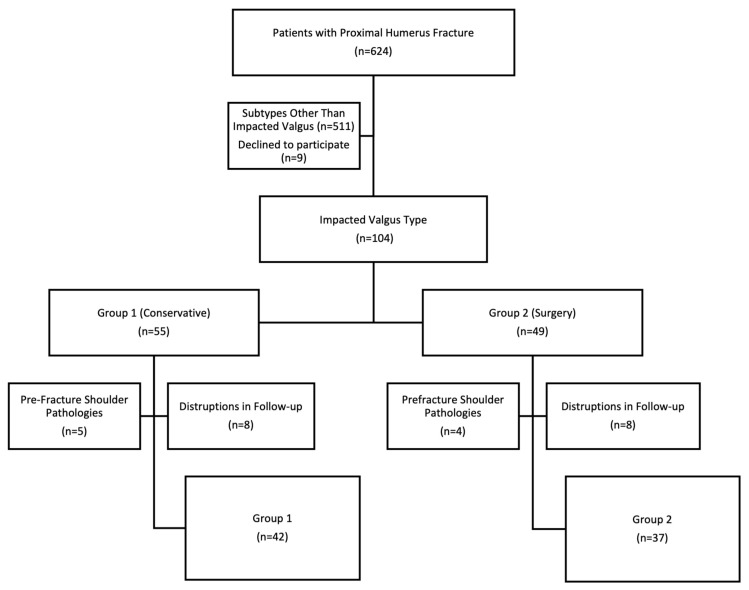
Flowchart of the study.

A total of 624 patients with proximal humerus fractures admitted to the orthopedic clinic between 2015 and 2021 were identified. A total of 113 patients were found to have an impacted valgus type fracture. A randomization approach for a retrospective study was implemented to reduce bias in treatment modality and patient selection. A random number was generated for each patient within each of the treatment modalities. The patients were ordered according to this random number, and study participation was solicited. Thus, a first come, first serve method was implemented. Nine patients declined to participate. The participants were divided into two groups, conservative (Group 1) and surgical (Group 2), according to the treatment modality, and the number of patients in the groups was 55 and 49, respectively. The treatment method of the patients was planned considering the morphology of the fracture, patients’ age, functional expectations, and activity level. Five patients from Group 1 and 4 patients from Group 2 were excluded from the study due to prefracture shoulder pathologies. After exclusion of patients with disruptions in follow-up (death or loss to follow-up), 42 conservatively and 37 surgically treated patients constituted the study population (Figure 1).

Deltopectoral incision, open reduction, and internal fixation with locking plates were applied to all patients in Group 2 with the same technique. After the surgery, the patients were given an arm sling. A Velpeau bandage was applied to Group 1.

### 2.1. Patient assessment

The American Shoulder and Elbow Surgeons Shoulder Score (ASES) and Constant Shoulder Score were obtained from the data that had been recorded during the first examination after treatment. The patients were evaluated for active and passive ROM (flexion (FLEX)), extension (EXT), abduction (ABD), adduction (ADD), internal rotation (IR), and external rotation (ER) after treatment. The patients’ pre- and posttreatment X-rays and CT scans were analyzed. Some of the radiographic parameters, including tubercle displacement (TD), cephalodiaphyseal angle (CDA), medial hinge (MH), cephaloglenoid angle (CGA), and medial hinge impaction (MHI), were measured by the same orthopedic surgeon. The follow-up time was a minimum of 24 months.

### 2.2. Radiological assessment

X-ray and CT scans were obtained in all patients before and after treatment. The shoulders of both groups were evaluated with a General Electric Medical Systems, LLC, Optima CT 660. (The arm was fixed to the patient’s body in the anatomical position. The reference lines were placed parallel to the clavicle with the glenoid at a right angle.) The slice thickness was 1.25 mm. To ensure the accuracy of measurements, a radiologic evaluation was performed by two authors who were experienced in musculoskeletal system imaging and who used two different picture archiving and communication systems (PACS); these authors were blinded to each other and to patient names. Both authors used OsiriX MD (Pixmeo, Bernex, Switzerland). The measurements obtained by the two authors were subjected to interobserver testing. The correlation between the two authors was evaluated by the interclass correlation coefficients (ICCs) from replicability analyses. Agreement was considered excellent if the ICC was >0.80, very good if it was 0.70–0.80, good if it was 0.60–0.70, fair if it was 0.40–0.60, and poor if it was <0.40. The interobserver alpha value was 0.91.

#### 2.2.1. Measurements

##### Tubercle displacement

**Figure 2:**
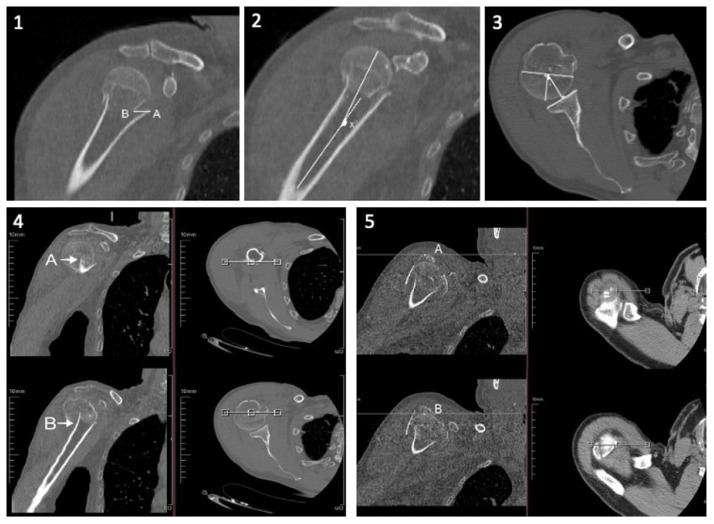
Measurement of parameters on computed tomography (1-medial hinge, 2-cephalodiaphyseal angle, 3-cephaloglenoid angle, 4-medial hinge impaction, 5- tubercle displacement).

This is the distance between the horizontal line drawn from the highest point in the section where the tuberculum majus is seen at the highest point in the coronal plane and the horizontal line drawn from the highest point of the articular surface of the humeral head in the section where the articular surface of the humeral head is seen at the highest point in the coronal plane [8] (Figure 2).

##### Cephalodiaphyseal angle

This is the angle between an imaginary line that is perpendicular to the humeral anatomical neck and extends to the tip of the humeral head in the section where the humeral head is most spherically seen in the coronal CT plane and an imaginary line that runs along the humeral diaphysis and is in the middle of the medulla in the coronal CT plane where the humeral diaphysis is seen widest [8] (Figure 2).

##### Medial hinge

**Figure 3:**
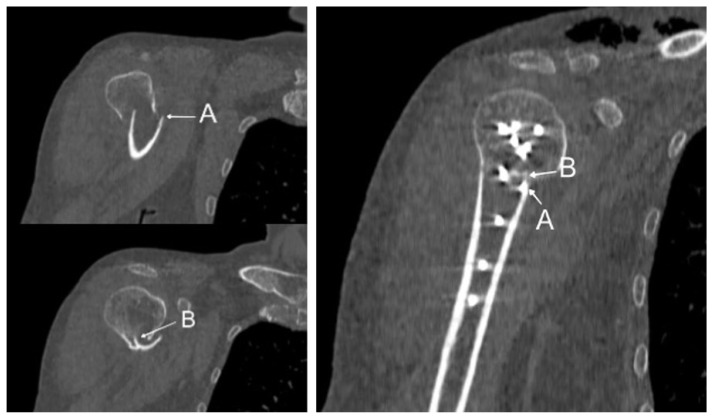
Pre- and postoperative measurement of the medial hinge on computed tomography (measured as the distance between the medial cortex in the section where the most proximal end of the distal part (diaphysis) of the fracture is seen widest in the coronal plane and the medial cortex of the most distal end of the proximal part in the section where the proximal part (humeral head) is seen most spherically in the coronal plane).

This is the distance between the medial cortex in the section where the most proximal end of the distal part (diaphysis) of the fracture is seen widest in the coronal plane and the medial cortex of the most distal end of the proximal part in the section where the proximal part (humeral head) is seen most spherically in the coronal plane (Figures 2 and 3).

##### Cephaloglenoid angle

This is the angle between a line drawn from the anterior end of the glenoid to the posterior end of the glenoid in the cross section where the glenoid articular surface is widest as seen in the axial CT plane and the line descending perpendicular to the glenoid articular surface from the middle of the line forming the anatomical neck of the humerus in the axial CT plane [9] (Figure 2).

##### Medial hinge impaction

**Figure 4:**
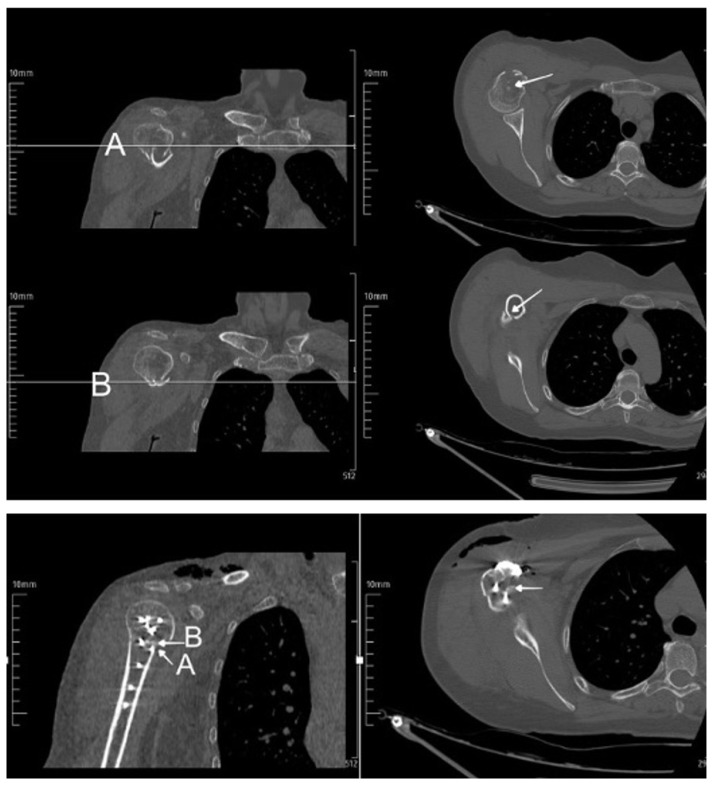
Pre- and postoperative measurement of medial hinge impaction on computed tomography (measured as the distance between the projections of the topmost slice, where the distal part (diaphysis) of the fracture is seen in the axial plane, and the lowest slice, where the proximal part (humeral head) of the fracture is seen in the axial plane, on the mapped coronal slice).

This is the distance between the projections of the topmost slice, where the distal part (diaphysis) of the fracture is seen in the axial plane, and the lowest slice, where the proximal part (humeral head) of the fracture is seen in the axial plane, on the mapped coronal slice (Figures 2 and 4).

### 2.3. Statistical analysis

NCSS software (Number Cruncher Statistical System, 2007, Utah, USA) was used for all analyses. Frequencies and percentages were calculated for demographic data, and comparisons of these data were performed using independent-samples t-tests and chi-squared tests. Means and standard deviations were reported for each measurement and each group. The Kolmogorov-Smirnov test was used to determine normality. Improvements in each period between the groups and differences between the pretreatment values and each follow-up period between the groups were analyzed using independent-samples t-tests and Mann-Whitney U tests. The Spearman correlation test was used to determine the relationships between variables and p < 0.05 was considered significant. The sample size was calculated using G*Power 3 (Heinrich Heine Universität Düsseldorf, Germany). Based on the calculations, a minimum sample size of 34 patients was required for each group to observe a correlation between the Constant values of the study and control groups [type 1 error (α) of 0.05, power (1 − β) of 0.80].

## 3. Results

A total of 79 patients met the inclusion criteria and were enrolled in the study. There was no statistically significant difference between the groups in terms of demographic variables (Table 1). The patients were evaluated in terms of clinical outcomes and radiological results.

### 3.1. Clinical outcomes

In the postoperative period, the ASES and Constant scores of the patients in Group 2 were significantly higher than those of the patients in Group 1 (Table 2). Additionally, passive EXT, active IR, and active and passive ER parameters were higher in Group 2 than in Group 1 (p values are 0.02, 0.03, <0.01, <0.01, respectively) (Table 3). There was no significant difference between the groups in other ROM parameters (Table 3).

### 3.2. Radiological results

#### Tubercle displacement

The reduction in tubercle displacement in both groups was statistically significant in the pre- and posttreatment periods. When intergroup TD was evaluated in the pretreatment period, it was observed that the mean value of Group 2 was higher than that of Group 1. However, there were no significant differences between the groups in the posttreatment period (Table 4).

#### Cephalodiaphyseal angle

The CDA measurements of the patients in Groups 1 and 2 were lower in the posttreatment period. Similar results were determined between the groups after treatment. However, the patients in Group 2 had CDA results higher than those of the patients in Group 1 before treatment (Table 4).

#### Medial hinge

For all patients in both groups, a statistically significant reduction in MH was defined after follow-up. The patients in Group 2 had higher MH results than those in Group 1 before treatment but lower results posttreatment (Table 4).

#### Cephaloglenoid angle

Both groups showed a reduction in the cephaloglenoid angle with treatment. The reduction was higher in Group 2 than in Group 1 (Table 4).

#### Medial hinge impaction

While the means of MHI were higher in Groups 1 and 2 before treatment, a significant improvement was observed after treatment. This improvement was higher in the patients treated surgically (Table 4).

### 3.3. Correlation between clinical and radiological outcomes

The Spearman correlation test showed that all radiological parameters investigated were significantly correlated with shoulder scores (ASES and Constant). Additionally, ASES and Constant scores were correlated with each other. When the relationship between functional results and radiological results was examined, it was observed that the strongest correlation was between MHI, MH, and functional results, considering the r values (Table 5).

## 4. Discussion

The primary finding of the present study is that radiological reduction of MH and MHI produces better functional outcomes in patients with impacted valgus proximal humerus fracture. Proximal humerus fracture is very common, particularly in elderly patients. Previously described classifications have guided surgeons in deciding on treatment for years [9,10]. However, it has been demonstrated that the described classifications have not included all fracture types. Furthermore, they could not provide adequate information on some fracture types, such as impacted valgus fracture [10,11].

Fracture type, age, sex, dislocation, patient activity level, and comorbid conditions are major factors in determining prognosis and treatment in proximal humerus fractures. However, prognostic factors have not been described for impacted valgus fractures. In the last decade, it has been shown that this fracture represents a spectrum of injuries [1]. Moreover, the efficacy of bone quality, arm position at injury, energy of trauma, vascular status, implant used, and radiological parameters have been investigated in the management of these fractures [10,12].

The management of impacted valgus fractures remains controversial [13]. Although a study showed significantly better bone quality in impacted valgus fractures, nonoperative treatment is recommended in elderly patients with severe morbidity and high perioperative risks [10,13]. Open reduction internal fixation is mostly recommended in individuals in good medical condition, owing to the satisfactory results obtained after surgery and the low risk of avascular necrosis. Anatomical reduction of the fracture results in good clinical outcomes and avoidance of secondary osteoarthritis [13]. In the present study, the treatment decision was made considering the patients’ consent and comorbid conditions.

Impacted valgus fracture differs from other fractures with its unique anatomy [1]. Radiological parameters have been used to define fracture geometry, treatment, and prognosis. Although there is ambiguity about the radiological definition of this fracture, in most studies it has been stated that the characteristic features are impaction of the humeral head in the metaphyseal region and a cervicodiaphyseal angle of more than 160° [1,14]. The inclusion criteria were also cervicodiaphyseal angle greater than 160° and impaction in the metaphyseal region in the present study. Additionally, MH and MHI were examined for the integrity of the posteromedial cortex. CGA and TD measurements were performed to specify the fracture geometry.

In the literature, the progression of fracture deformity in the conservative treatment of proximal humerus fracture was investigated, and it was emphasized that fracture deformity increased with standard conservative treatment. Moreover, it was observed that the mean reduction in the valgus tilt of the articular surface–cervicodiaphyseal angle and tuberosity displacement were not statistically significant after treatment compared to the initial deformity. As a result, it was asserted that conservative treatment did not significantly improve radiological parameters in proximal humerus fractures [15]. However, in the present study, both CDA and TD were reduced after conservative or surgical treatment. This mean reduction was statistically significant after both treatments.

Hertel et al. investigated the relationship between humeral head perfusion and radiological outcomes. It has been shown that the integrity of the medial hinge and anatomical neck and calcar extension are strongly associated with humeral head perfusion. Angular displacement of the head and the amount of TD were reported to be poor predictors for humeral head ischemia [11]. Regardless of vascular status, the integrity of the medial hinge is an important support in fracture reduction and fixation. It has been emphasized that the medial hinge should be reduced first in patients with severe lateral displacement [11,16]. In the current study, medial hinge reduction was the primary purpose in patients treated surgically to preserve humeral head ischemia.

Filling the space in the impacted area with a graft in surgical treatment is one technique applied. However, its indications for impacted valgus fractures are variable and depend on the surgeon’s preference [7]. It has been observed that there was no redisplacement when tubercle reduction and medial hinge continuity were provided in impacted valgus fractures. Further, it has been emphasized that functional outcomes were good in patients who met these requirements (tubercle reduction, medial hinge continuity), so the use of graft in patients with defects is not an absolute necessity [17].

In staging impacted valgus fractures based on radiological parameters and soft tissue status, it has been suggested that tubercle fractures should be treated according to their own characteristics [9]. In addition, it has been shown that the medial periosteal hinge was ruptured with an average lateral head displacement of 3.5 mm, and all soft tissue was disrupted [18]. It has been suggested that surgical treatment should be the first-line treatment in young patients with good activity levels and these radiological features [19].

There is little information in the literature regarding how the amount of displacement of each fragment in impacted valgus fractures affects functional outcomes. In one study, more than half of valgus-impacted fractures showed worsening in functional outcomes. It has been stated that the height of the greater tuberosity affects the pain scores the most. A 0.8-point worsening in the pain score was observed for each 10 mm superior location of the greater tuberosity related to the articular surface [9]. In the present study, radiological healing was observed in all parameters after treatment. Moreover, in the correlation analyses between these parameters and functional outcomes, it was determined that radiological improvement had a positive effect on clinical results. In this context, MH and MHI were the most effective parameters for clinical outcomes.

## 5. Limitations

The study has a number of limitations. First, it is subject to the typical biases associated with its retrospective design, such as sampling bias. In addition, as previously described, a large number of patients were lost to follow-up. These patients, who may have obtained significant benefit or very minor benefit, may be more likely to respond to postoperative surveys and have the potential to skew our findings. An additional limitation is that the radiological measurements were not homogeneous between the groups in the pretreatment period. However, it is inevitable that fractures with worse radiological data are in the surgical group. The strengths of our study were interobserver evaluation and randomization to prevent bias.

## 6. Conclusion

Impacted valgus proximal humerus fractures remain a controversial topic. It is necessary to evaluate the fracture geometry together with patient-related factors in their management. According to the findings of the present study, medial hinge integrity and amount of impaction are highly influential on functional outcomes. While planning treatment, these parameters should be carefully monitored. In addition, the most important goal of treatment should be correction of these parameters. In cases in which these parameters are within acceptable limits or can be corrected with conservative treatment, surgery is not the unique and absolute option.

## Figures and Tables

**Table 1:** Demographic variables of the patients.

	Group 1	Group 2	p value
**Patients**	42	37	
**Age, years**	65.3 ± 16.9 (24–98)	60.5 ± 12.7 (29–83)	0.08 ¥
**Male/female sex**	10/32 (23.8%/76.2%)	13/24 (35.1%/64.9%)	0.26 Ψ
**Right/left side**	12/30 (28.6%/71.4%)	11/26 (29.7%/70.3%)	0.91 Ψ
**Smoker/nonsmoker**	6/36 (14.3%/85.7%)	8/29 (21.6%/78.4%)	0.39 Ψ
**Duration of union, weeks**	8 ± 0.8 (8–10)	8 ± 2.1 (8–12)	0.08 ¥
**Duration of arm sling use, weeks**	5.9 ± 0.9 (4–7)	7.6 ± 3.2 (4–12)	0.09 ¥

NOTE. Data are presented as the mean ± standard deviation (range) or number of patients.

Ψ Chi-squared test,

¥ Independent-samples t-test

**Table 2:** Shoulder scores of the patients (postoperative period).¥

Parameter	Group 1	Group 2	p value
**ASES score**	68 ± 14.5	90.2 ± 6.1	<0.01*
**Constant score**	66.5 ± 14.1	88.7 ± 5.6	<0.01*

NOTE. Data are presented as the mean ± standard deviation.

* p < 0.05,

¥ Independent-samples t*-*test

ASES: American Shoulder and Elbow Surgeons.

**Table 3:** Range of motion parameters of the patients (postoperative period).¥

Parameter	Group 1	Group 2	p value
**Active FLEX**	160 ± 36.5	167.1 ± 21.4	0.33
**Passive FLEX**	167.1 ± 25.6	170 ± 22.4	0.41
**Active EXT**	51.4 ± 9	44.4 ± 15.1	0.15
**Passive EX**	57.1 ± 4.9	51.4 ± 3.8	0.02*
**Active ABD**	142.9 ± 41.9	145.7 ± 30.5	0.44
**Passive ABD**	157.1 ± 30.4	162.9 ± 21.4	0.34
**Active ADD**	37.1 ± 4.9	37.1 ± 9.5	0.5
**Passive ADD**	33.6 ± 8.5	39.3 ± 9.3	0.13
**Active IR**	35.7 ± 7.9	52.6 ± 20.6	0.03*
**Passive IR**	45.7 ± 9.8	54.3 ± 21.5	0.17
**Active ER**	50 ± 11.5	74.3 ± 16.2	<0.01*
**Passive ER**	64.3 ± 7.9	80 ± 11.5	

NOTE. Data are presented as the mean ± standard deviation.

* p < 0.05,

¥ Independent-samples t-test

FLEX: flexion; EXT: extension; ABD: abduction; ADD: adduction; IR: internal rotation; ER: external rotation.

**Table 4:** Radiological measurements performed in computed tomography for each period.

Measurement variable	Pretreatment	Posttreatment	p value
**TD (mm)**			
**Group 1**	2.4 ± 1.7	1.6 ± 1.5	0.04 * ¥
**Group 2**	4.9 ± 3.3	1.7 ± 1.8	<0.01* ¶
**Group 1 vs. Group 2**	p < 0.01* ¥	p = 0.09 ¶	
**CDA (°)**			
**Group 1**	171.5 ± 12.3	159.6 ± 13.3	<0.01* ¥
**Group 2**	183.8 ± 13.2	159.8 ± 14.3	<0.01* ¥
**Group 1 vs. Group 2**	p < 0.01* ¥	p = 0.9 ¥	
**MH (mm)**			
**Group 1**	4.3 ± 2.2	2.6 ± 1.4	<0.01* ¶
**Group 2**	7.3 ± 4.3	1.6 ± 1.1	<0.01* ¶
**Group 1 vs. Group 2**	p < 0.01* ¶	p < 0.01* ¶	
**CGA (°)**			
**Group 1**	−20.7 ± 11.4	−15.7 ± 6.4	0.01* ¥
**Group 2**	−31.9 ± 13	−15.6 ± 5.7	<0.01* ¶
**Group 1 vs. Group 2**	p < 0.01* ¶	p = 0.7 ¶	
**MHI (mm)**			
**Group 1**	4.9 ± 1.6	3.6 ± 1.9	<0.01* ¶
**Group 2**	6.9 ± 2.5	1.9 ± 1.8	<0.01* ¶
**Group 1 vs. Group 2**	p < 0.01* ¶	p < 0.01* ¶	

NOTE. Data are presented as the mean ± standard deviation.

* p < 0.05,

¥ Independent-samples t-test,

¶ Mann-Whitney U test

TD: tubercle displacement; CDA: cephalodiaphyseal angle; MH: medial hinge; CGA: cephaloglenoid angle; MHI: medial hinge impaction

**Table 5:** Correlation between functional outcomes and radiological measurements.

		ASES	Constant
**ASES**	r		0.92
	p		<0.01*
**Constant**	r	0.92	
	p	<0.01*	
**TD (mm)** **Pre-/Posttreatment difference**	r	0.27	0.28
	p	0.02*	0.01*
**CDA (°)** **Pre-/Posttreatment difference**	r	0.37	0.35
	p	<0.01*	<0.01*
**MH (mm)** **Pre-/Posttreatment difference**	r	0.71	0.68
	p	<0.01*	<0.01*
**CGA (°)** **Pre-/Posttreatment difference**	r	−0.39	−0.38
	p	<0.01*	<0.01*
**MHI (mm)** **Pre-/Posttreatment difference**	r	0.83	0.82
	p	<0.01*	<0.01*

* p < 0.05
